# Navigating features: a topologically informed chart of electromyographic features space

**DOI:** 10.1098/rsif.2017.0734

**Published:** 2017-12-06

**Authors:** Angkoon Phinyomark, Rami N. Khushaba, Esther Ibáñez-Marcelo, Alice Patania, Erik Scheme, Giovanni Petri

**Affiliations:** 1 ISI Foundation, Turin 10126 Italy; 2 Institute of Biomedical Engineering, University of New Brunswick, Fredericton, New Brunswick, Canada E3B 5A3; 3 Faculty of Engineering and Information Technology, University of Technology, Sydney, New South Wales 2007, Australia; 4 Indiana University Network Institute, Indiana University, Bloomington, IN, USA

**Keywords:** topological data analysis, topological simplification, feature extraction, feature selection, myoelectric control, electromyogram, EMG

## Abstract

The success of biological signal pattern recognition depends crucially on the selection of relevant features. Across signal and imaging modalities, a large number of features have been proposed, leading to feature redundancy and the need for optimal feature set identification. A further complication is that, due to the inherent biological variability, even the same classification problem on different datasets can display variations in the respective optimal sets, casting doubts on the generalizability of relevant features. Here, we approach this problem by leveraging topological tools to create charts of features spaces. These charts highlight feature sub-groups that encode similar information (and their respective similarities) allowing for a principled and interpretable choice of features for classification and analysis. Using multiple electromyographic (EMG) datasets as a case study, we use this feature chart to identify functional groups among 58 state-of-the-art EMG features, and to show that they generalize across three different forearm EMG datasets obtained from able-bodied subjects during hand and finger contractions. We find that these groups describe meaningful non-redundant information, succinctly recapitulating information about different regions of feature space. We then recommend representative features from each group based on maximum class separability, robustness and minimum complexity.

## 1. Introduction

Biological pattern recognition systems are finding a growing number of applications, such as computer-aided diagnosis for breast cancer [1], prosthesis control [2] and brain–computer interfaces [3]. Great progress has been made using deep learning techniques when large amounts of labelled data are available [4]. In applications for which limited data are available and full deep learning is not yet viable, it is crucial to be able to identify optimal feature sets for classification and analytical purposes [5]. Biological signals, however, are complex and vary within and across subjects, making the selection of feature sets that are reusable across subjects and tasks a highly non-trivial task. Indeed, it is known that the feature sets yielding the best performances can change between very similar classification problems or datasets [6]. One should therefore exercise caution when interpreting the optimal feature set for practical use in biological pattern recognition.

To navigate this problem in a tangible way, we focus on a specific case study, electromyogram (EMG) signals, for which feature selection is a current and acknowledged problem in the scientific literature. Specifically, Kamavuako *et al.* [7] reported that there is no consensus on the optimum threshold value of the two most commonly used EMG features, zero crossings and slope sign changes [8], leading them to investigate the effect of threshold selection on classification performance, on robustness over time and on the ability to generalize across multiple datasets. The results showed that the optimum threshold (when the minimum error rate was found for classification of hand motions) is highly subject and dataset dependent. That is, each subject had a unique optimum threshold value, and, even within the same subject, the optimum threshold could change over time (i.e. the subject-dependent optimum thresholds do not generalize well).

In practical use, one desires models that are trained on data from one set of users and able to classify another [9], and also training data recorded over a few days that can then be used on subsequent days [10]. Therefore, Kamavuako *et al.* [7] recommended a global optimum threshold value yielding a good trade-off between classification performance and generalization (i.e. the one that yielded the global minimum classification error rate across subjects and datasets). In consideration of the above, we propose a novel strategy to navigate this problem by reducing the exploration of feature spaces to that of a simplified topological chart [11], which can effectively guide the feature selection process, avoiding redundancy in a human-parsable and principled way. Specifically, we adopt topological methods to determine functional groups of EMG features across subjects and datasets, as opposed to using traditional feature selection algorithms to automatically select a subject-and-data-dependent optimal feature set. The resulting principled and robust map of feature space can be used as a guideline to choose a global optimal feature set that exhibits a good trade-off between classification performance and generalization.

We first identify sub-groups and functional groups of 58 state-of-the-art EMG features by applying a topology-based data analysis method called Mapper [11]. The Mapper method can identify unique and relevant sub-groups in several research problems that classical clustering and dimensionality reduction methods (e.g. single-linkage hierarchical cluster analysis (HCA) and principal component analysis (PCA)) cannot detect [12,13]. We then select representative features for these groups based on three properties: maximum class separability, robustness and complexity [14,15]. Based on results from previous studies, it was hypothesized that features selected using Mapper can achieve at least the same level of classification accuracy as when using all features [16]. To validate this hypothesis and the generality of the results, we provide a comparison of Mapper and the commonly used feature selection algorithm, sequential forward selection (SFS), for classification of hand and finger contractions.

## 2. Methodology

### 2.1. Electromyographic datasets

Three EMG datasets [17–19] collected independently at different institutes were used. There was no prior coordination of data acquisition methods and experimental protocols. The same data preprocessing methods implemented in the original works were reproduced. Thus between the datasets, the recorded and preprocessed EMG data differ from one another. While performance of EMG features presumably depends on the dataset, multiple datasets are necessary to examine the robustness and generalization of the findings [7,20].

In the first dataset, the EMG data were recorded from four forearm muscles in twenty normally limbed subjects, as they performed eight motions: hand open, hand close, wrist flexion, wrist extension, wrist radial deviation, wrist ulnar deviation, forearm pronation and forearm supination. The subjects were asked to perform three sessions per day for four separate days and in each session they performed five trials (60 trials in total). Within each trial, the subject performed each motion for 2 s in duration and separated each motion by a 2 s rest period. The order of motions was randomized in each session. All EMG signals were amplified with a gain of 19.5 × and sampled at 1024 Hz with a 24-bit resolution (Mobi6-6b, TMS International B.V.). The EMG data were passed through a band-pass filter with a cut-off frequency of 20 and 500 Hz and a notch filter with a cut-off frequency of 50 Hz. In total, 1920 2-s EMG data (60 trials × 8 motions × 4 muscles) were collected for each subject (for more details, see [17]).

In the second dataset, the EMG data were recorded from seven forearm muscles in 30 normally limbed subjects, as they performed six motions: hand open, hand close, wrist flexion, wrist extension, forearm pronation and forearm supination. The subjects were asked to perform four sessions and in each session they performed six trials (24 trials in total). Throughout a trial, four repetitions of 3 s were collected for each motion, as well as four random rest periods. All EMG signals were amplified with a gain of 1000 × and sampled at 3000 Hz (Model 15, Grass Telefactor). The EMG data were passed through a band-pass filter (10–400 Hz) and resampled from 3000 to 1000 Hz. In total, 4032 3-s EMG data (24 trials × 4 repetitions × 6 motions × 7 muscles) were collected for each subject (for more details, see [18]).

In the third dataset, the EMG data were recorded from two forearm muscles in eight normally limbed subjects, as they performed 10 motions: the flexion of each of the individual fingers (i.e. thumb, index, middle, ring, little), the pinching of combined thumb–index, thumb–middle, thumb–ring, thumb–little and hand close. The subjects were asked to perform six trials per motion for a period of 5 s. The order of motions was randomized with random resting periods between motions. All EMG signals were amplified with a gain of 1000× (Bagnoli-8, Delsys) and sampled at 4000 Hz with a 12-bit resolution (BNC-2090, National Instruments). The EMG data were passed through a band-pass filter (20–450 Hz) and a notch filter (50 Hz) as well resampled from 4000 to 1000 Hz. In total, 120 5-s EMG data (6 trials × 10 motions × 2 muscles) were collected for each subject (for more details, see [19]).

### 2.2. Electromyographic pattern recognition

The EMG signals are typically used to assess muscle activation by measuring electrical activity in muscles using multiple surface electrodes. One of the most important applications is the use of forearm EMG signals as a control signal for prosthetic hands, referred to as ‘myoelectric control’ [2]. Recent developments in low-cost commercial products of wireless and wearable EMG devices (e.g. Myo armband) have provided an opportunity to use forearm EMG signals as inputs for ‘muscle–computer’ interfaces [21].

After preprocessing of the raw EMG signals, EMG pattern recognition systems typically include four main components: data segmentation, feature extraction, classification and controller [2]. The procedure includes projecting the signals to a lower dimensional space, where the dimensions represent features. A classifier then recognizes signal patterns and classifies them into pre-defined classes, i.e. hand and finger motions in this study.

Indeed, many previous studies have shown that the success of EMG pattern recognition systems mainly depends on the selection of high-quality features [8,10]. Importantly, in addition to maximum class separability, a high-quality feature set should also display good robustness and minimal complexity [14,15]. For instance, low-computational resource devices may require features with low-computational complexity and higher degree of robustness against noise. During the last three decades, a wealth of EMG feature extraction techniques has been proposed and applied to hand and finger motion classification (e.g. [8,20,22]). In spite of the success achieved, however, many EMG features are highly correlated, raising the issue of feature set redundancy. Surprisingly, there have been few systematic studies comparing EMG features, especially from a redundancy viewpoint [23].

Fifty-eight feature extraction methods in both the time and frequency domains [10,20,22] were applied to surface EMG signals from the three datasets described above. Full names and abbreviations are shown in table 1 with their specific parameter values used for each dataset as well as the references for mathematical definitions. It should be noted that some feature extraction techniques provided more than one feature value. As a result, 81 individual EMG features were extracted. Furthermore, feature scaling was performed using standardization (per EMG channel and per subject) so that features have zero mean and a unit variance. This preprocessing step was used for further algorithms applied in this study, i.e. to have features on the same scale for PCA (as it emphasizes features on larger measurement scales), and to equalize the contribution of all features to *k*-nearest neighbours analysis with a Euclidean distance measure.

**Table 1.** A list of EMG feature extraction techniques.

full names	abbreviations	parameters	dimensions	references
amplitude of the first burst	AFB	*w*_f_ = 32 ms	1	[23]
approximate entropy, sample entropy	ApEn, SampEn	*m* = 2, *r* = *σ* × 0.2	1, 1	[10,24]
autoregressive model and its differencing version	AR, DAR	order = 4	4, 4	[14,22,25]
box counting dimension	BC	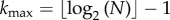	1	[10,26]
cepstrum/cepstral coefficients and its differencing version	CC, DCC	order = 4	4, 4	[22,25]
critical exponent analysis	CEA	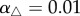	1	[27,28]
difference absolute mean value	DAMV	—	1	[22,25,29]
difference absolute standard deviation value	DASDV	—	1	[22,29]
detrended fluctuation analysis	DFA	*n*_*i*_ = 2^*i*^, *i* = 2 − 6, order = 2	1	[17,30]
maximum-to-minimum drop in power density ratio	DPR	—	1	[31,32]
frequency ratio	FR	*f*_lb_ = [20 45], 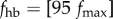	1	[23,33,34]
Higuchi's fractal dimension	HG	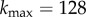	1	[17,35]
histogram	HIST	segment = 3	3	[14,23]
integrated EMG	IEMG	—	1	[14,23,25]
Katz's fractal dimension	KATZ	—	1	[10,36]
kurtosis, skewness	KURT, SKEW	—	1, 1	[37,38]
log detector and its differencing version	LD, DLD	—	1, 1	[14,22,23]
The second-order moment	M2	—	1	[20,22]
mean absolute value	MAV	—	1	[8,14,39]
modified mean absolute value (type 1, type 2)	MAV1, MAV2	—	1, 1	[23,34]
mean absolute value slope	MAVS	segment = 2	1	[8,23]
maximum amplitude	MAX	cut-off = 5 Hz, order = 6	1	[10,40]
median frequency, mean frequency	MDF, MNF	—	1, 1	[41,42]
maximum fractal length	MFL	—	1	[10,35]
multiple hamming/trapezoidal windows	MHW, MTW	—	3, 3	[23,43]
mean power, total power	MNP, TTP	—	1, 1	[10,23,43]
myopulse percentage rate	MYOP	threshold = 20/0.02/5 × 10^−5^	1	[10,23]
power spectrum deformation	OHM	—	1	[31,32]
peak frequency	PKF	—	1	[10,23]
power spectral density fractal dimension	PSDFD	—	1	[10,44]
power spectrum ratio	PSR	*n* = 20	1	[23,45]
root mean square	RMS	—	1	[23,39]
spectral moment	SM	order = 2	1	[23,43]
signal-to-motion artefact ratio, signal-to-noise ratio	SMR, SNR	—	1, 1	[31,32]
slope sign change	SSC	threshold = 16/10^−4^/10^−10^	1	[8,23]
simple square integral	SSI	—	1	[23,43]
time-dependent power spectrum descriptors	TDPSD	—	6	[20]
absolute temporal moment and its differencing version	TM, DTM	order = 3	1, 1	[22,23]
variance and its differencing version	VAR, DVARV	—	1, 1	[14,22,23]
variance of central frequency	VCF	—	1	[23,43]
variance fractal dimension	VFD	—	1	[10,46]
*v*-order and its differencing version	V, DV	order = 3	1, 1	[14,22,23]
Willison amplitude	WAMP	threshold = 20/0.02/5 × 10^−5^	1	[14,23]
waveform length	WL	—	1	[8,23]
zero crossing	ZC	threshold = 10/0.01/10^−5^	1	[8,14,23]

### 2.3. Functional groups of electromyographic features

Topological data analysis (TDA) is an approach that focuses on extracting and understanding the ‘shape’ of data using techniques from topology (for an introduction and a survey, see [47,48]). This set of tools allows for extraction of relevant insights from complex data with high-dimensionality, high-variability, low signal-to-noise ratio, time-dependence and nonlinearity [49]. In particular, a topological simplification method called Mapper was employed in this work [11]. The basis of the Mapper method is to produce controlled simplifications of the data by means of a series of local clustering in overlapping regions of the data space and by successively linking together clusters that share common data points. The process to compute Mapper is composed of four steps.

(1) *Transforming raw data into a point cloud*: in the current study a point cloud was a set of EMG features in the original high-dimensional space or the reduced lower-dimensional space (using a PCA approach). The 81 EMG features comprised the rows of the matrix for all the datasets. For the first dataset, the columns comprised of either the entire set of 38 400 (1920 2-s EMG data × 20 subjects) feature values or the first 28 principal component (PC) scores (explaining 95% of the total variance in the raw feature vector). Both were included in this work to enable a comparison of their respective performances. Only the reduced PC-dimensional space explaining 95% of the total variance was involved for the second (the first 29 PCs) and the third (the first 24 PCs) datasets.
(2) *Segmenting the point cloud data into *MI* intervals overlapped with percentage *MO* using one or more filter functions*: in this study the Euclidean distance to the *k*th nearest neighbour (*k*-NN distance) was used as a filter function. Note that the first nearest neighbour (*k* = 1) of a data point is always the point itself, so the second nearest neighbour (*k* = 2) was implemented to measure the distance from any data point to the nearest data point other than itself [50].
(3) *Applying any standard clustering algorithm to create clusters from each sub-dataset corresponding to the defined intervals*: in this study, Ward's minimum variance method was employed as a criterion for the HCA [51]. The clusters defined in the intervals become the nodes of a topological network. The number of features in each node is indicated using the size of the node and the number in it.
(4) *Constructing the topological network*: connecting pair of nodes that share data points across adjacent intervals of the filter functions, i.e. the edges of a topological network.

As a result, the nodes of the topological network can be considered as sub-groups of EMG features, the edges and their strengths represent the presence and strength of the overlap between clusters (shown by the thickness of the edge). To make these sub-groups more interpretable, ‘a functional group’ is defined as a collection of similar Mapper nodes belonging to coherent sections of the topological network (e.g. long linear segments) and displaying similar mathematical definitions as well as information contained in the features.

### 2.4. Selection of representative features

The topological EMG feature sub-groups can be interpreted using the following guidelines.

(1) Features in network nodes with low *k*-NN distance (or low filter values) are highly correlated to others in the same sub-group. In other words, the cluster is ‘stronger’ and better defined. This suggests that one should select only one feature (or few) from each of these sub-groups.
(2) Although features with high *k*-NN distance (more independent features) can be locally clustered into sub-groups (weaker clusters), several EMG features from these sub-groups can be selected as they should contain different types of information.

To identify representative (or relevant) features from each functional group, two widely used feature evaluation methods, separability measures and classifiers, were employed for within-subject pattern classification [14,15,33,34]. A feature vector was created for each of the datasets, subjects and features, and used as an input for the evaluation methods. Specifically, the number of columns is equal to the number of muscles and the number of rows is the number of the analysis segments. For instance, the dimensions of the matrix for each of the subjects and features in the first dataset with the segment length of 250 ms and the segment increment of 125 ms are 7200 × 4 (for details on data segmentation, see [52]). The influence of the data segmentation on classification was also investigated.

(1) *Using separability measures*: evaluating feature space based on statistical criteria. In this study, the Davies–Bouldin index (DBI) [53] and Fisher's linear discriminant analysis index (FLDI) were employed [54]. DBI is obtained by averaging the worst-case separation of each class from the others. Instead of considering only the worst situation distances, FLDI considers all classes together which is defined as the ratio of the within-class scatter matrix to the between-class scatter matrix. Lower values of statistical indices (the lowest value is 0) imply a higher degree of class separability.
(2) *Classifying the feature space*: imposing classification boundaries on features and measuring classification error rates (or misclassification rates) using a 10-fold cross-validation technique. In this study, linear discriminant analysis (LDA) and support vector machine (SVM) approaches were employed. Multiclass SVM [34] was implemented using the one-against-one method with a linear kernel [55]. An error rate is defined as the number of incorrect classifications divided by the total number of test samples and then multiplied by 100. Lower error rates imply a higher degree of class separability (the lowest possible rate is 0%).

### 2.5. Effective electromyographic feature sets

To demonstrate the power of the topological feature chart in designing effective sparse feature sets, three well-known EMG feature sets were re-evaluated by replacing redundant features from the same functional group with more relevant features from that group (i.e. minimizing overlap and maximizing class separability).

(1) *TD feature set*: MAV, WL, ZC, SSC and MAVS [8];
(2) *AR+RMS feature set*: AR and RMS [56,57];
(3) *AR+CC+WL feature set*: AR, CC and WL [58];

Further, to validate the effectiveness of the topological chart as a guide for feature selection, a comparison of the Mapper approach [11] and the more common SFS [59,60] was conducted. For Mapper, a global optimal feature set was chosen by collecting representative features from each of the functional groups. For SFS, a sequential search across subjects using 10-fold cross-validation method of within-subject misclassification rates was performed and features were selected for each dataset using 70% of the data (a training set). The performance of the selected features from Mapper and SFS was then compared to the baseline performance with all features using the remaining (30%) of the data from the same set (the test set) as well as a 10-fold cross-validation for the other dataset. The Cohen's *d* effect size [61], defined as the difference between two group means divided by a standard deviation, was used to report the meaningful differences between classification error rates of the different feature sets [62]. For Cohen's *d*, an effect size of 0.2 equates to a small effect, 0.5 equates to a medium effect and larger than 0.8 equates to a large effect [61].

## 3. Results

### 3.1. Functional groups of electromyographic features

**Figure 1.**
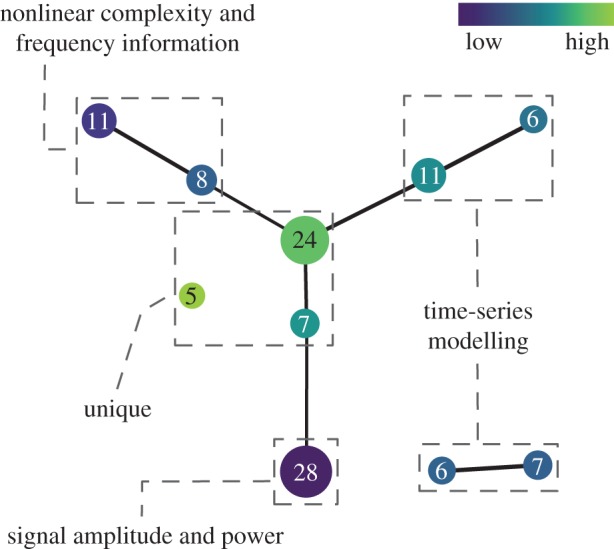
The resulting topological network computed using three intervals with a 50% overlap from the 28 PC scores extracted from the first dataset. *k*-NN distance was used as a filter function. The colours encode the filter values, with blue indicative of low distance, and green of high. The number of features in each node is indicated using the size of the node and the number in it.

**Figure 2.**
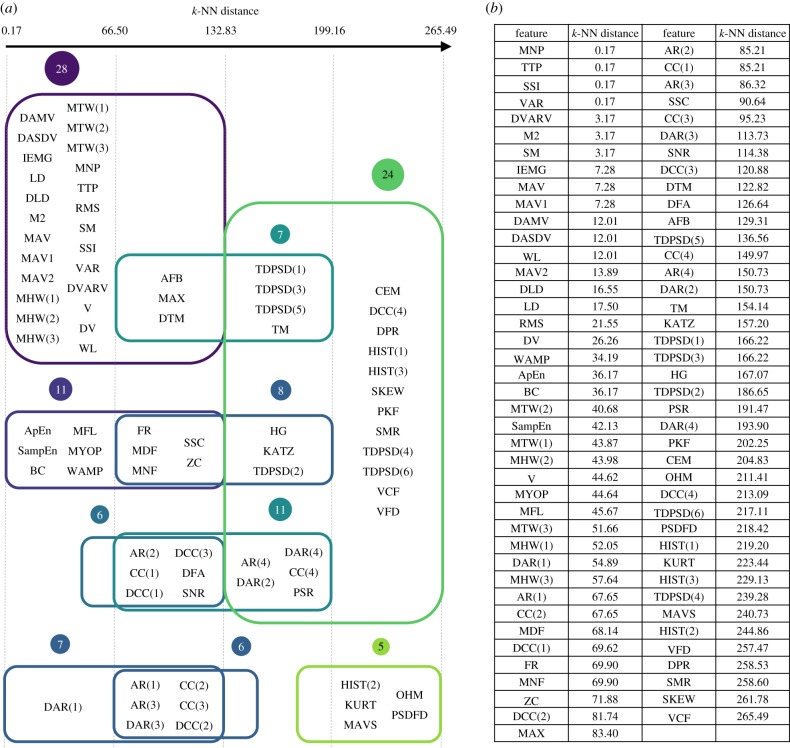
Sub-groups of EMG features (*a*) derived from the topological network of the first EMG dataset and their corresponding filter values (*b*) sorting from smallest to largest. *k*-NN distance was used as a filter function. The colours encode the filter values, with blue indicative of low distance, and green of high.

The topological network computed using MI = 3 intervals overlapped with MO = 50% from the 28 PC scores for the first EMG dataset is shown in figure 1. A complete list of features in each node can be found in figure 2. The resulting topological network consists of 10 nodes and has a main structure shaped like the letter Y composed of three arms connected to a central core, along with two additional components disconnected from the main structure. Using the network shape together with the nature of the information contained in the features, four functional groups were defined as follows.

(1) The lower arm consisted of features that are used to estimate signal magnitude and power (e.g. MAV and RMS).
(2) The upper left arm consisted of features that contain frequency information (e.g. ZC and MDF) and features that are used to measure the nonlinearity and complexity of time series (e.g. entropy and fractal dimensions).
(3) The upper right arm and the two disconnected nodes consisted of time-series modelling features (e.g. AR).
(4) The central core and a single disconnected node depict a set of features that provide unique information (e.g. HIST and MAVS).

**Figure 3.**
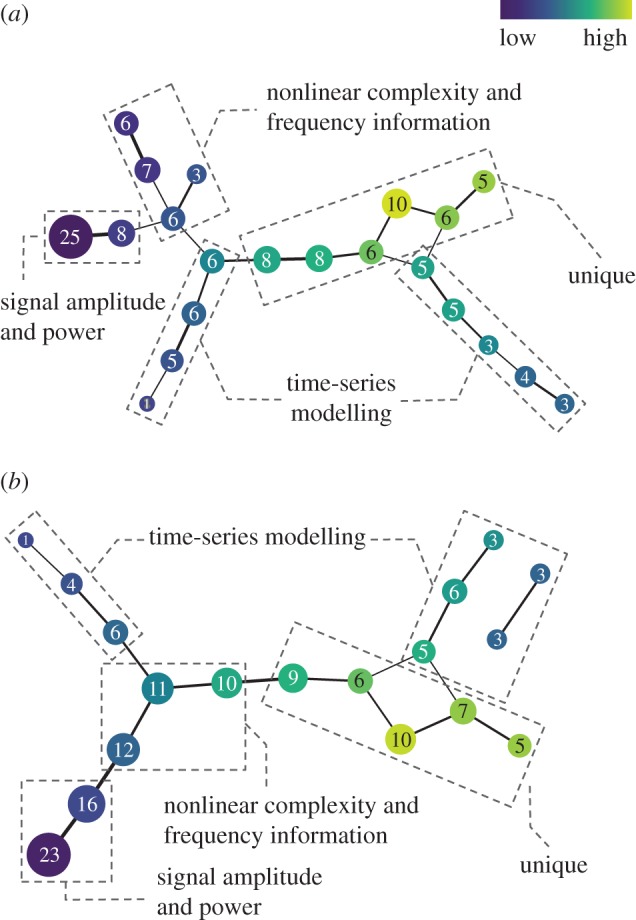
The resulting topological networks computed using eight intervals with a 50% overlap from (*a*) the reduced lower-dimensional space (28 PC scores) and (*b*) the original high-dimensional space (38 400 feature values) for the first dataset. *k*-NN distance was used as a filter function. The colours encode the filter values, with blue indicative of low distance, and green of high.

**Figure 4.**
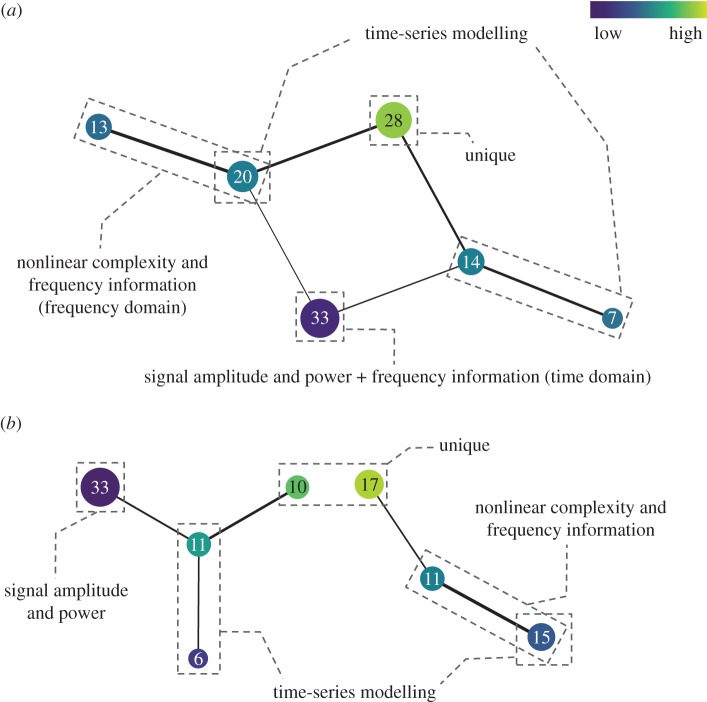
The resulting topological networks computed using three intervals with a 50% overlap from the reduced lower-dimensional space for (*a*) the second dataset (29 PC scores) and (*b*) the third dataset (24 PC scores). *k*-NN distance was used as a filter function. The colours encode the filter values, with blue indicative of low distance, and green of high.

Figure 3 shows the results obtained by applying Mapper to the same dataset (the first dataset), but, using different Mapper parameters and input types. By comparing figure 3*a* with figure 1, we find that, while more intervals produced more nodes (or sub-groups), the shape of the resulting topological networks is similar. EMG features conveying similar information still cluster together. The same findings were obtained even when the dimensions of the point cloud data were extended from 28 to 38 400 (figure 3*b*), as well as in the second (figure 4*a*) and third datasets (figure 4*b*). These results confirm the robustness of the proposed method and the generality of the determined groups.

### 3.2. Selection of representative features

The results of the feature evaluation methods are summarized in table 2. For the signal amplitude and energy feature group, WL, DAMV and DASDV, which are the differential versions of the well-known time-domain features (IEMG, MAV and RMS), could be used as representative features. Features in the nonlinear complexity and frequency information feature groups with strong discrimination power included MFL, SampEn and WAMP. In the time-series modelling feature group, the differential versions of the AR and CC features provided slightly better classification performance than their original counterparts. Although the individual predictive power of features in the unique feature group was lower than features in other feature groups, the classifiers performed considerably better than chance for many features (e.g. TDPSD and HIST), suggesting that there exists potential for improving the classification performance when combined in a feature set. It is intuitive that when the segment length was decreased, the misclassification rate increased (see results in the electronic supplementary material). However, due to real-time constraints (a segment increment plus the processing time of feature extraction and classification approaches should be less than 300 ms [52]), short segment lengths and increments are necessary.

**Table 2.** The classification performance of 81 features using different evaluation methods: DBI, FLDI, SVM and LDA for the three EMG datasets.

	data 1				data 2	data 3		data 1				data 2	data 3
feature	DBI	FLDI	SVM	LDA	SVM	LDA	feature	DBI	FLDI	SVM	LDA	SVM	LDA
AFB	3.42	9.92	42.7	50.6	54.3	43.1	MAX	3.08	8.07	31.3	40.0	42.5	30.6
ApEn	1.63	1.74	11.7	15.8	38.8	23.1	MDF	2.66	4.73	33.2	36.0	20.8	31.7
SampEn	1.79	1.82	14.7	17.2	39.9	26.9	MNF	2.37	4.21	27.0	31.0	18.0	26.5
AR(1)	2.34	4.75	28.4	32.2	15.4	22.7	MFL	1.69	1.86	10.6	14.2	2.7	12.9
AR(2)	2.55	6.01	34.6	37.7	15.6	22.3	MHW(1)	2.51	4.66	22.5	33.7	27.9	29.6
AR(3)	2.70	6.43	40.0	40.6	21.3	28.1	MHW(2)	2.50	4.74	23.6	36.5	13.1	31.5
AR(4)	3.31	8.77	48.1	48.5	33.5	32.3	MHW(3)	2.69	6.72	26.6	38.2	13.0	30.0
DAR(1)	2.34	4.44	28.2	31.9	13.0	22.1	MTW(1)	2.18	3.58	19.1	29.5	27.2	25.4
DAR(2)	2.75	7.18	39.9	40.8	19.5	26.9	MTW(2)	2.39	4.19	21.7	33.7	12.8	25.4
DAR(3)	2.46	4.13	33.7	35.6	16.1	25.2	MTW(3)	2.56	5.36	24.6	36.4	13.5	31.0
DAR(4)	2.94	7.20	45.2	45.3	22.3	35.2	MNP	2.18	3.43	18.8	29.4	21.9	25.2
BC	1.83	2.32	18.4	21.2	43.0	36.9	TTP	2.18	3.43	19.1	29.2	21.9	26.2
CC(1)	2.34	4.75	28.6	31.9	15.1	22.3	MYOP	1.94	2.87	15.8	20.5	8.2	13.1
CC(2)	2.53	5.66	33.1	36.1	14.8	23.1	OHM	3.00	8.35	39.8	44.3	22.6	27.3
CC(3)	2.90	8.33	37.7	42.0	17.1	19.8	PKF	5.65	21.31	66.2	66.5	46.2	62.3
CC(4)	3.22	11.96	44.6	47.4	32.4	21.0	PSDFD	3.48	9.67	48.2	49.5	29.3	62.5
DCC(1)	2.34	4.44	28.4	32.0	12.7	22.7	PSR	4.78	16.93	59.1	60.2	40.8	52.5
DCC(2)	2.64	6.02	37.2	38.3	15.5	22.9	RMS	2.03	2.60	15.6	21.5	16.6	19.2
DCC(3)	2.52	4.16	33.8	36.0	16.3	32.1	SM	1.93	2.43	16.0	26.5	9.9	26.9
DCC(4)	2.94	7.02	44.2	44.7	21.4	34.6	SMR	15.83	218.21	86.8	83.6	36.9	66.5
CEA	4.05	10.21	54.0	54.7	76.0	65.2	SNR	3.12	10.87	39.9	45.1	30.9	22.5
DAMV	1.87	1.89	11.9	18.7	4.1	20.6	SSC	1.82	2.46	17.9	21.7	3.6	30.2
DASDV	1.80	1.88	11.9	18.0	5.0	22.9	SSI	2.18	3.43	18.6	29.3	21.7	25.0
DFA	2.28	4.25	27.9	30.4	17.4	26.9	TDPSD(1)	3.61	3.27	39.9	39.0	15.3	27.1
DPR	12.13	184.14	81.7	83.0	79.2	60.6	TDPSD(2)	2.48	3.07	27.2	29.0	13.7	17.1
FR	2.72	4.56	36.0	39.4	17.1	36.2	TDPSD(3)	2.54	2.67	31.8	36.3	18.2	38.1
HG	4.29	14.19	48.3	52.4	33.2	39.6	TDPSD(4)	2.17	4.81	25.9	29.5	15.7	27.5
HIST(1)	6.32	35.20	40.5	50.5	39.1	51.2	TDPSD(5)	2.84	3.37	35.1	35.6	18.8	26.0
HIST(2)	4.42	19.28	32.0	39.0	31.9	48.3	TDPSD(6)	2.38	4.99	29.1	31.2	13.6	16.0
HIST(3)	6.28	40.45	40.7	50.4	40.2	48.5	TM	4.85	31.73	50.4	58.3	50.1	33.3
IEMG	2.03	2.53	14.7	21.4	10.8	18.5	DTM	3.64	11.32	38.0	48.6	30.4	40.4
KATZ	4.03	9.41	54.2	54.6	44.0	47.9	VAR	2.18	3.43	19.0	29.6	22.2	26.0
KURT	5.97	31.76	62.1	66.1	53.0	46.9	DVARV	1.95	2.48	16.2	26.5	9.8	26.5
SKEW	11.00	212.11	80.0	78.7	62.8	30.8	VCF	18.25	340.56	87.8	87.6	82.2	67.9
LD	2.47	3.08	19.6	24.8	8.2	20.0	VFD	13.08	156.55	83.4	83.0	71.5	65.8
DLD	2.26	2.59	19.2	23.9	3.7	20.2	V	2.00	2.85	17.4	22.8	20.7	19.8
M2	1.95	2.48	16.1	26.8	9.9	26.2	DV	1.81	1.97	13.1	18.5	6.5	20.6
MAV	2.03	2.53	14.6	21.2	10.9	17.9	WAMP	1.77	2.12	12.4	17.3	4.3	15.2
MAV1	2.05	2.61	14.9	21.7	9.6	18.3	WL	1.87	1.89	11.9	18.7	4.2	21.0
MAV2	2.11	2.69	15.6	22.4	8.4	19.6	ZC	1.77	1.89	17.6	20.9	5.1	27.5
MAVS	3.37	9.95	39.1	42.1	34.9	45.6							

### 3.3. Effective electromyographic feature sets

To re-design effective sparse feature sets based on the obtained topologies, the first and the third datasets were investigated. It is important to note that the findings were similar for both datasets.

(1) *TD feature set*: based on the first dataset, the best single feature was WL (16.85% error). The error rates were reduced to 11.56%, 9.82%, 9.56% and 9.59% when adding SSC, MAV, ZC and MAVS, respectively, into the feature set. A meaningful improvement (*d* = 0.71) was found when SSC was added into the feature set but no further meaningful differences (*d* = 0.01 − 0.29) were found when adding more features.
(2) *AR + RMS feature set*: based on the third dataset, the best individual feature was RMS (28.17% error). The error rates were reduced to 18.04%, 14.23%, 11.96% and 11.28% by adding AR(1), AR(2), AR(3) and AR(4), respectively. Meaningful improvements (*d* = 0.57 − 1.72) were found when adding AR(1), AR(2) and AR(3), but no difference (*d* = 0.18) was found when adding the last feature.
(3) *AR + CC + WL feature set*: using this feature set as an example to demonstrate how a feature set could be re-designed using the context provided by the Mapper approach, the original AR features could be replaced with their differential version, DAR. CC (which is in the same functional group as AR) could also be replaced with representative features from other functional groups (DAMV, DASDV, MFL and WAMP). With the same number of features, the classification error rate of the re-designed feature set using the third dataset decreased from 10.74% to 7.38% (*d* = 0.57).

To evaluate the effectiveness of using the topological feature chart to select an effective and generalizable feature set, we used representative features from each of the functional groups. A set of DAMV, DASDV, WAMP, ZC, SampEn, MFL, DAR(1–4) and TDPSD(1–6) (16 out of 81 EMG features) were chosen based on the extracted topologies and the feature performances for the first dataset shown in table 2. Owing to the fact that similar topological charts and functional groups were found and determined across different datasets, this set of features could be used as a global optimal feature set. As a comparison, SFS was constrained to using the same number of features as found using Mapper. The features selected using SFS for the first dataset consisted of DAMV, DLD, V, SSC, SampEn, ApEn, MFL, BC, PSDFD, DAR(1,2,4), AR(4), HIST(2) and SKEW. For the third dataset, the features selected were DASDV, DVARV, DTM, DV, WAMP, SSC, MYOP, MNF, ApEn, MFL, HG, DAR(1–4) and SKEW. The SVM misclassification rates for the first and the third datasets when using the Mapper selected features, the two sets of SFS selected features and the set of all features are presented in table 3. In addition to the classification performance, the total computational time was measured. Approximately 1 s was used by Mapper to create feature charts of any dataset in this study while the computational times for SFS on the first dataset (26880 2-s EMG data) and the third dataset (672 5-s EMG data) were approximately 6 h and approximately 49 min, respectively.

**Table 3.** The classification performance of the Mapper selected features, the SFS selected features and all features using the SVM classifier for the first and the third EMG datasets.

feature set	data 1: test set	data 3	feature set	data 3: test set	data 1
Mapper	4.95	7.62	Mapper	7.07^a^	4.70^a^
SFS (data 1: training set)	4.61	6.62	SFS (data 3: training set)	15.03^b^	11.01^b^
all features	5.24	6.15	all features	5.66	4.76

^a^Meaningful difference between Mapper and SFS (*d* > 0.8).

^b^Meaningful difference between SFS and all features (*d* > 0.8).

## 4. Discussion

### 4.1. Functional groups of electromyographic features

The proposed method was successful in identifying four distinct functional groups of EMG features across multiple EMG datasets with different data acquisition methods and experimental protocols. These results are also consistent with a previous study [23] that analysed a subset of the 58 features used in the present study.

(1) *The signal amplitude and power feature group*: the first and the biggest functional group determined. Features in this group captured the same kind of information, i.e. the signal magnitude.
(2) *The nonlinear complexity and frequency information feature group*: besides two types of nonlinearity and complexity measures of time series (e.g. SampEn, ApEn, MFL, BC), this functional group also included frequency information features which were composed of time-domain features (e.g. WAMP, ZC and SSC) and frequency-domain features (e.g. MNF, MDF and FR).
(3) *The time-series modelling feature group*: AR and CC. Both methods shared the same feature spaces [23] and achieved similar classification error rates (table 2). One should therefore select either AR or CC. These feature extraction methods can also provide features that belong to different sub-groups. For example, in the case of the AR model of order 4, the first- and the third-order AR coefficients were clustered into the 6- and 7-feature nodes while the second- and the fourth-order AR coefficients were clustered into the 6- and 11-feature nodes. Since these feature nodes have moderate-to-high *k*-NN distances, all the coefficients could be included in the feature vector.
(4) *The unique feature group*: features in this group captured different kinds of information from the EMG data. Most of the features in this group are an extension of features in other groups. For example, MAVS is the difference between two consecutive MAV features [8] and HIST is an extension of the ZC and WAMP features [14]. A number of relevant features from this group can be chosen.

Based on the current findings (which are consistent with previous multi-dataset studies) together with the strong theoretical foundation of TDA (which is a coordinate free approach [12]), it is reasonable to expect that these four functional groups of EMG features should be able to be applied to new EMG data. It is also important to note that to make it simple for interpretation, in this study the four functional groups were defined using topological feature charts with low-resolution parameters (figure 1). However, if the problem of interest is very complex and needs a high-dimensional feature set to achieve an acceptable classification accuracy, we can use a topological feature chart with a higher resolution (figure 3) and then select representative features from more feature sub-groups identified by Mapper without a pre-existing knowledge of the information in the features.

### 4.2. Selection of representative features

The results of the present investigation are in support of [22] and suggest that the classification performance of features extracted from the first difference of EMG time series (referred to here as differential versions) was better than their original counterparts derived directly from the windowed raw EMG. This is due to the fact that time-domain and frequency-domain feature methods are not designed to reliably quantify a non-stationary signal while EMG signal stationarity varies depending on the data segmentation and types of muscle contractions [63]. A differencing technique can be used to transform surface EMG signals such that they become more stationary. As a result, the within-class variation of features extracted from the transformed EMG signal decreases while the distance of clusters between motions is preserved [22]. These features existed in the signal amplitude and power feature group as well as the time-series modelling feature group, and thus could be used as the representative features in a configuration such as WL, DAMV, DASDV, DAR or DCC. It can also be observed that the classification accuracies achieved by DAMV (a differencing version of MAV) were often slightly higher than DASDV (a differencing version of RMS). This is likely due to the fact that, on average, the probability density function (PDF) of forearm EMG signals during upper-limb motions is closer to a Laplacian density, and both theory and experiment indicate that an optimal EMG amplitude estimator based on the Laplacian model is MAV [39]. Since the EMG PDF is dependent on many factors involving muscle locations, muscle contraction levels and types of motion, one can evaluate the correct identification of PDF shape of EMG using the robust measures of kurtosis [37]. In general, however, the selection of the differencing version of well-known EMG features should provide a better classification performance.

An interesting point for the nonlinear complexity and frequency information feature group is that it contains many robust EMG features. Specifically, WAMP can preserve the cluster separability in a noisy environment, both power line interference [64] and random noise [65]. WAMP also showed the best discriminant power among the frequency information features (i.e. ZC, SSC and MYOP). These features use a threshold to reduce the effect of background noise. The selection of the optimum threshold values is thus important not only for the classification performance [7] but also for the robustness [64,65]. Next, among 50 feature extraction methods proposed in Phinyomark *et al*. [10], SampEn is the most robust to systemic EMG signals changes over time (or the effect of between-day variation), followed by ApEn and MFL. These three features are all from the nonlinear complexity feature group. It should be noted that although it is possible to compute SampEn and ApEn on a digital signal processor chip embedded in the prosthetic hand palm [66], the computational complexity of the entropy methods is higher than other time-domain features proposed in this study.

Lastly, the most interesting feature in the non-redundant feature group is TDPSD. This feature set can reduce the impact of force level variations [20]. In support of their findings, although the individual discriminant power of each feature in the TDPSD feature set does not solely describe all the characteristics of the targeted motions (table 2), these features were found to have minimal redundancy. Thus, when these features are combined in one set, they should maximally cover the entire space of EMG signals associated with motions and improve robustness.

### 4.3. Effective electromyographic feature sets

It can be clearly observed from the TD and the AR+RMS feature sets that when the number of features fed into the classifier increased, the misclassification rate decreased. However, when the number of selected features from the TD feature set was more than two and more than four for the AR+RMS feature set, the misclassification rate showed a slight decrease. This is because features from similar groups provide insufficient novel information to overcome the added dimensionality that they impose (the curse of dimensionality).

In support of our hypothesis, the Mapper selected feature set achieved the same level of classification error rate as the exhaustive set of features. Similar performance was also found using the SFS approach on the first dataset, however, this came at a tremendous computational cost of approximately 21 600 times greater based on ranking 81 features across 20 subjects. More importantly, while the performance of this feature set generalized to the third dataset, the opposite case was did not. The SFS feature set selected using the third dataset failed to provide the same classification performance when it was tested using the first dataset (11.01% error rate instead of 4.61%) while the Mapper selected feature set provided the same classification performance across datasets. These results suggest that the more generative understanding of feature types obtained from topological feature charts could lead to a better design of a globally generalizable feature set, and be less prone to over-tuning than purely data-driven methods such as SFS.

In summary, the benefits of using the determined functional groups for feature selection can be realized in three ways: (i) with the same number of features, we can replace features that have low discriminant power with ones from the same group, which may provide similar types of information but better discrimination. We can expect the new feature set to be more representative of the targeted motions, therefore leading to better classification performance; (ii) equivalently, we can use a smaller feature set to effectively cover the same space that a larger feature set does (such as reduce the 81 feature set to the selected 16 feature set). In this case, 65 redundant and/or irrelevant features were removed without reducing the classification performance; and (iii) the understanding provided by the topologies can enable pattern recognition system designers to incorporate additional prior knowledge, leading to more robust and generalizable feature selection. Furthermore, the design of novel features could be informed by these topologies. New features should ideally cluster into the unique feature group, or even lead to the creation of new groupings.

### 4.4. Limitations and future studies

A first limitation is that time-frequency or time-scale transformations (e.g. discrete wavelet transform) were not included, as their derived values have not directly been used as EMG features [67,68]. Dimensionality reduction (e.g. MAV [15,69] or PCA [67,70]) methods are necessary to apply to them, making their interpretation less intuitive. Their performance also depends on many factors such as mother wavelet and decomposition level. For this reason, in this study we chose to investigate only time and frequency domain features to simplify the interpretation of the results. Future studies investigating the range of time-frequency analysis features would be a valuable additional to the literature. Second, we did not investigate the effect of feature parameters such as the threshold values of ZC, SSC, WAMP and MYOP or the order of AR and CC. In the present investigation, feature parameters were determined based on previous recommendation in the literature and/or preliminary studies. The effect of feature parameter selection on the feature space and classification performance should be investigated further. Third, we did not include EMG data recorded from amputees in the analysis. Although the relationship between surface EMG signals acquired from intact-limbed subjects and amputees has not been clearly defined yet, Hargrove *et al.* [71] evaluated the effects of the seven feature sets for able-bodied and amputee subjects, and found that the same trend appeared between them. Together with the generalization of the current findings across multi-EMG datasets from normal subjects, it is reasonable to expect that the functional groups of EMG features defined should be able to be applied to EMG pattern recognition control of multi-functional prostheses by amputees. Fourth, in this study, we focus on investigating EMG features for myoelectric prostheses and muscle–computer interface. Thus, only forearm surface EMG signals acquired from sparse multi-EMG channels during hand and finger contractions were investigated. Future research should consider applying the proposed techniques in other EMG-related research problems such as high-density EMG, gait analysis, speech recognition or detecting neuromuscular abnormalities. Finally, we acknowledge that there will be new feature extraction methods proposed in the research community, and thus the re-evaluation of new EMG features and of their position in the topological feature map will be necessary.

## 5. Conclusion

In this work, we presented an application of topological simplification techniques to explore multi-dimensional feature space and provide a topological chart able to identify four functional groups of EMG features and the relationships between them. Representative features from each group were selected based on their classification ability, enabling the intuitive and generative design of effective sparse feature sets. We further showed that the proposed topological chart is robust and generalizes well across multiple datasets, when compared with purely data-driven feature selection techniques such as SFS. These results support the usefulness of clustering- and feature selection-based topological networks for improving both the performance and understanding of EMG-based pattern recognition. In this contribution, we focused on a specific type of biological signals. However, the approach described here can be directly generalized to any other type of biological signals displaying complex patterns of correlations among features, such as electroencephalogram data [72], functional magnetic resonance imaging data [73] or biomechanical data [74], in which the dimensionality of the data space is typically much larger than the volume of the data itself.

## Supplementary Material

Classification errors rates for dataset 1

## Data accessibility

Datasets 1 and 2 can be requested from the corresponding authors of [17] and [18]. Dataset 3 can be accessed via http://www.rami-khushaba.com/electromyogram-emg-repository.html.
