# Supplementary material for: Navigating features: a topologically informed chart of electromyographic features space
Source: J R Soc Interface. 2017 Dec 6;14(137):20170734. doi: 10.1098/rsif.2017.0734 (PMC5746577; doi:10.1098/rsif.2017.0734)
Supplement: Classification errors rates for dataset 1 [file rsif20170734supp1.pdf]

Navigating features: a topologically-informed chart of EMG features space

Angkoon Phinyomark[1,2], Rami N. Khushaba[3], Esther Ibáñez-Marcelo[1], Alice Patania[4], Erik Scheme[2], and Giovanni Petri[1,*]

1 ISI Foundation, Turin 10126 Italy
2 Institute of Biomedical Engineering, University of New Brunswick, Fredericton, New Brunswick, Canada E3B 5A3
3 Faculty of Engineering and Information Technology, University of Technology, Sydney, New South Wales 2007, Australia
4 Indiana University Network Institute, Indiana University, Bloomington, IN, USA

* giovanni.petri@isi.it

Supplementary File

The results showed that when the segment length decreased, the misclassification rate increased. However, due to real-time constraints that a segment increment plus the processing time of feature extraction and classification approaches should be less than 300 ms, small segment length and increment are necessary. Based on the results obtained from the first dataset, for example, the increase in the error rate of WL was 2.3% and 5% when the segment length-increment decreased from 2000-2000 ms to 500-125 ms and 250-125 ms, respectively. Some EMG features are highly sensible to the data segmentation. For example, the increase in the error rate of ZC was 19.4% when the segment length-increment reduced from 2000-2000 ms to 250-125 ms. The misclassification rates obtained from the first EMG dataset using the SVM classifier for all features when the segment length and increment changed from the whole movement (2 s) to between 125 and 500 ms are shown in Table 1.

Table 1. The classification error rates obtained from the first EMG dataset using the SVM approach when the segment length and increment were changed from the whole movement (2-s) to between 125-500 ms or (128-512 samples).

Feature	Segment length-increment			Feature	Segment length-increment			Feature	Segment length-increment		
	512-128	256-128	256-256		512-128	256-128	256-256		512-128	256-128	256-256
AFB	37.6	37.9	39.7	HIST(1)	39.2	42.4	43.2	OHM	47.9	55.8	56.4
ApEn	32.3	47.1	48.8	HIST(2)	33.7	37.2	38.1	PKF	68.4	71.0	71.3
SampEn	16.6	22.1	25.1	HIST(3)	39.0	42.2	43.1	PSDFD	60.3	68.6	69.3
AR(1)	38.4	47.7	49.2	IEMG	18.3	22.1	23.0	PSR	64.1	69.4	70.0
AR(2)	47.6	57.7	58.8	KATZ	59.2	62.5	63.7	RMS	19.4	23.0	24.0
AR(3)	53.4	63.6	64.4	KURT	72.3	75.3	75.9	SM	16.8	19.8	20.9
AR(4)	59.8	68.4	69.1	SKEW	78.4	80.5	81.2	SMR	87.5	87.5	87.5
DAR(1)	36.1	44.1	45.6	LD	20.0	24.1	25.3	SNR	57.4	68.9	70.0
DAR(2)	50.7	59.5	60.3	DLD	16.7	19.5	20.8	SSC	28.6	37.9	39.7
DAR(3)	44.5	54.2	55.3	M2	17.2	20.0	21.4	SSI	22.8	26.8	28.2
DAR(4)	56.5	65.4	66.2	MAV	18.3	22.0	23.0	TDPSD(1)	47.6	55.4	56.4
BC	36.8	48.4	50.0	MAV1	18.5	22.5	23.6	TDPSD(2)	35.8	45.0	45.9
CC(1)	38.4	47.7	49.3	MAV2	18.7	22.7	23.8	TDPSD(3)	41.6	49.0	50.3
CC(2)	46.0	56.2	57.4	MAVS	75.9	86.6	87.6	TDPSD(4)	35.0	43.1	44.1
CC(3)	52.6	62.7	63.8	MAX	43.6	45.0	45.0	TDPSD(5)	43.2	52.8	53.9
CC(4)	58.2	66.7	67.4	MDF	42.2	51.1	52.2	TDPSD(6)	39.4	50.2	51.2
DCC(1)	36.0	44.0	45.6	MNF	33.8	41.9	43.3	TM	53.7	56.4	57.7
DCC(2)	47.2	56.2	57.3	MFL	13.5	16.1	16.8	DTM	40.6	45.1	47.0
DCC(3)	44.0	53.7	54.9	MHW(1)	32.5	39.3	40.4	VAR	22.7	26.8	28.2
DCC(4)	55.6	64.6	65.5	MHW(2)	29.6	38.6	40.6	DVARV	17.2	20.1	21.3
CEA	66.8	73.2	73.4	MHW(3)	28.3	38.1	39.9	VCF	87.8	87.7	87.6
DAMV	14.2	16.8	17.8	MTW(1)	27.5	32.4	33.2	VFD	83.9	83.9	84.3
DASDV	14.7	17.4	18.2	MTW(2)	25.0	30.6	32.1	V	21.0	24.4	25.4
DFA	35.5	44.0	45.6	MTW(3)	24.4	30.9	32.8	DV	15.9	18.6	19.4
DPR	82.0	84.0	83.9	MNP	22.7	26.8	28.2	WAMP	16.3	20.7	22.0
FR	45.9	55.0	55.8	TTP	22.8	26.8	28.2	WL	14.2	16.9	17.7
HG	63.8	65.4	67.4	MYOP	21.3	26.8	28.2	ZC	27.9	37.0	38.8
